# An On-chip Spiking Neural Network for Estimation of the Head Pose of the iCub Robot

**DOI:** 10.3389/fnins.2020.00551

**Published:** 2020-06-23

**Authors:** Raphaela Kreiser, Alpha Renner, Vanessa R. C. Leite, Baris Serhan, Chiara Bartolozzi, Arren Glover, Yulia Sandamirskaya

**Affiliations:** ^1^Institute of Neuroinformatics, University of Zurich and ETH Zurich, Zurich, Switzerland; ^2^Lincoln Centre for Autonomous Systems, University of Lincoln, Lincoln, United Kingdom; ^3^Istituto Italiano di Tecnologia, Genoa, Italy

**Keywords:** pose estimation, event-based vision, neuromorphic SLAM, on-chip learning, scene memory, iCub robot, visual reset, spiking neural networks

## Abstract

In this work, we present a neuromorphic architecture for head pose estimation and scene representation for the humanoid iCub robot. The spiking neuronal network is fully realized in Intel's neuromorphic research chip, Loihi, and precisely integrates the issued motor commands to estimate the iCub's head pose in a neuronal path-integration process. The neuromorphic vision system of the iCub is used to correct for drift in the pose estimation. Positions of objects in front of the robot are memorized using on-chip synaptic plasticity. We present real-time robotic experiments using 2 degrees of freedom (DoF) of the robot's head and show precise path integration, visual reset, and object position learning on-chip. We discuss the requirements for integrating the robotic system and neuromorphic hardware with current technologies.

## 1. Introduction

Neuromorphic hardware implements the non-Von Neumann brain-inspired computing architecture based on known properties of biological neural networks. This computing architecture features event-based asynchronous processing and fine-grained parallelism of a network of spiking neurons (Indiveri et al., 2009; Schemmel et al., 2010; Furber et al., 2012; Merolla et al., 2014; Galluppi et al., 2015; Qiao et al., 2015; Davies et al., 2018; Moradi et al., 2018). Neuromorphic hardware not only supports parallel processing; it also enables feedback loops, recurrence, and online adaptation—the key properties of biological brains that lead to flexible and robust behavior. Biological neural systems evolved to solve tasks that are highly relevant to robotics: perception, movement control, action planning, or decision making under uncertainty. Thus, robotics is a promising application domain for neuromorphic hardware (Krichmar and Wagatsuma, 2011). Autonomous robots require that computing be performed with low latency and low power consumption, and these are the key characteristics of neuromorphic devices. In this work, we contribute to the emerging field of neuromorphic robotics by presenting a number of design patterns—spiking neural network models—to solve one of the key robotic tasks, state estimation.

To be used efficiently, neuromorphic hardware requires a radical rethinking of the computing paradigm. In neuromorphic hardware, we cannot run functions, create conditional loops, or have if-then-else statements in the same way as in conventional software. To use the brain-inspired computing substrate—neurons and synapses—efficiently, we need to abandon the notion of addition and multiplication as elementary computing operations. Even the mere representation of values as binary bit-strings becomes obsolete in a neuronal computing framework. Instead, neuromorphic systems represent the measured physical variables and perform computation using events (spikes) spreading in a neuronal network, as brains do. Thus, in our work, we aim to develop the neuronal computing elements that are required to solve different tasks, seeking to derive principles and structures that can be reused in different domains. Moreover, we show how an interface can be established between the sensors and motors of a robot and neuromorphic representations.

Neuronal network-based algorithms currently deliver the most impressive results in computer vision and machine learning and are increasingly being deployed in robotics (Chen et al., 2015; Mnih et al., 2015). Training spiking neuronal networks (SNNs) using methods developed for deep learning (i.e., error backpropagation) is challenging and currently leads to reduced performance compared to conventional, full-precision DNNs (Shrestha and Orchard, 2018; Neftci et al., 2019). On the other hand, the neuromorphic hardware supports online learning, i.e., the adaptation of synaptic weights after deployment of the system. When designing our SNN models, we do not rely on tabula-rasa data-driven learning. Instead, we leverage the knowledge of neuronal circuits that solve similar tasks in animals, reserving learning only to parts that depend on the environment with which the robot interacts. This learning can happen in a “shallow” network.

Findings in neuroscience have inspired a number of neuronal architectures for addressing the problem of simultaneous localization and mapping (SLAM) (Arleo and Gerstner, 2000; Cuperlier et al., 2007; Barrera and Weitzenfeld, 2008; Weikersdorfer et al., 2013; Milford and Schulz, 2014; Jauffret et al., 2015). SLAM is one of the core problems in mobile robotics (Stachniss et al., 2016) but can be generalized to any robotic system that requires state estimation of the robot relative to its environment. In this work, we present a spiking neural network (SNN) implemented on Intel's neuromorphic research chip, Loihi, for pose estimation of the robot's head. The pose is estimated in the SNN based on the “efferent copy” of the motor commands. The estimate is corrected by a visual cue when the robot sees an object multiple times during exploration of an environment. The initial pose, under which the object was seen the first time, is learned in plastic synapses on chip and is used for the visual reset. Estimating the pose by integrating the motor commands is referred to as dead reckoning in robotics and as path integration in biology.

In robotics, the head-pose estimation amounts to the camera pose estimation problem (e.g., Scaramuzza and Fraundorfer, 2011). The camera pose is estimated using on-board sensors to measure an incremental change in pose, e.g., the visual system itself, a built-in inertial measurement unit (IMU), laser range finder, time of flight camera (Engelhard et al., 2011) or sonar (Thrun et al., 2007). Fusion of information from multiple sensors is performed to provide a more robust estimate of the pose change. Since integration of movement is prone to error accumulation, such systems need frequent recalibration. The global positioning system (GPS) or external cameras, e.g., Vicon system, help to avoid this problem, but in many cases measuring the ground-truth pose directly is not possible, and the problem becomes one of simultaneous localization and mapping (SLAM). The reference relative to which the pose is measured is itself estimated concurrently with the estimate of the pose (Stachniss et al., 2016).

Animals can also navigate in large environments by combining a set of “on-board” sensors, i.e., the vestibular and vision system (Burak and Fiete, 2009; Seelig and Jayaraman, 2015; Green et al., 2017; Fisher et al., 2019). They combine motion commands and internal sensing in their neuronal systems to provide a motion estimate. Even simple animals, such as insects, show complex navigation behaviors. A brain region called the central complex (CX) appears to be their navigation center (Pfeiffer and Homberg, 2014; Turner-Evans and Jayaraman, 2016; Heinze, 2017). Visual landmarks (Seelig and Jayaraman, 2013), rotational optic flow, and non-visual angular velocity cues (Green et al., 2017; Turner-Evans et al., 2017) were shown to mediate direction coding in CX neurons, suggesting that allothetic and idiothetic cues are continuously integrated to generate a robust representation of body orientation (Honkanen et al., 2019). The orientation appears to be encoded in the activity bump of neurons arranged in a ring that corresponds to the 360° of possible directions. These computational principles were uncovered in brains with a size of <100K neurons and were shown to fit small-scale neuromorphic platforms (Dalgaty et al., 2018).

Orientation-selective Head Direction (HD) cells have also been discovered in rodents. Several models propose attractor networks to account for their selective firing behavior (Skaggs et al., 1995; Redish et al., 1996). Such attractor networks might self-organize to respond best to the observed sensory information (Stringer et al., 2002). These models have been mapped onto brain anatomy, explaining which brain regions might be involved in the encoding of angular velocity signals, the current head direction estimate, and the update mechanism (Goodridge and Touretzky, 2000). The detailed mapping of the HD neuronal circuits gave rise to a Spiking Neural Network (SNN) model in which persistent activity is realized through cross-inhibition rather than through recurrent excitation, as previously assumed (Song and Wang, 2005). The function of the HD network is to act as a neural integrator that is supervised by visual signals (Hahnloser, 2003) and supposedly is calibrated through angular velocity signals (Stratton et al., 2010). The vestibular information appears to be critical for generating the directional signal, and landmark information is important for updating it (Taube, 2007).

Inspired by the biological findings regarding navigation systems of insects and mammals, several computing architectures have been developed to estimate the position under uncertainty and re-calibrate it using familiar landmarks (Skaggs et al., 1995; Samu et al., 2009; Arena et al., 2013; Erdem et al., 2015; Seelig and Jayaraman, 2015; Heinze et al., 2018). An early successful attempt of a bio-inspired SLAM was the RatSLAM model—a biologically inspired SLAM system able to map indoor and outdoor environments (Milford et al., 2004). Recently, the original RatSLAM model was extended to function in 3D environments (Yu et al., 2019). Loop closure detection was realized based on visual template matching (Gu and Yan, 2019), and multi-sensor fusion was shown to provide more accurate odometry and precise cognitive mapping (Zhang et al., 2019). Neural networks of grid cells have been shown to perform long-range navigation through path integration in the 2-dimensional plane (Edvardsen, 2017), and a model that was established through the Neural Engineering Framework confirms the attractor map implementation of path integration and proposes that the head direction signal can be used to modulate allocentric velocity input (Conklin and Eliasmith, 2005).

These computational approaches are complemented by approaches toward neuromorphic SLAM, which realized neuronal models in neuromorphic hardware. In this line of research, the formation of a 1D-map was demonstrated on a neuromorphic chip that could perform Bayesian inference using path integration and visual estimate (Tang et al., 2019). A model of the bat navigational system was realized in a neuromorphic VLSI device (Massoud and Horiuchi, 2012). This architecture includes a head direction ring attractor network (Massoud and Horiuchi, 2011a) and online correction through learned landmarks that are identified using sonar sensory signals (Massoud and Horiuchi, 2011b). Similarly, our previous work on neuromorphic SLAM, implemented on a miniature autonomous vehicle, incorporates a 1D head direction ring, 2D map formation, and a loop closure detection mechanism (Kreiser et al., 2018b,c, 2019a) based on vision. A neuromorphic system that can generate angular velocity and linear acceleration using IMU signals can be used as input to an HD network and was implemented on a VLSI chip to model the vestibular system (Corradi et al., 2014). More recently an SNN model was proposed for performing angular velocity regression on event-based visual data (Gehrig et al., 2020) that could potentially be used as input to an HD network when implemented in neuromorphic hardware.

Up until now, current approaches to neuromorphic implementations have been proofs of concept and either have not been deployed in a real-world scenario using a robotic agent or do not address the issue of scaling and performance under disturbances. In this work, we build on previous implementations for orientation estimation and use the biologically inspired head-direction network (Seelig and Jayaraman, 2015; Green et al., 2017; Fisher et al., 2019) to build an SNN model that estimates the pose of the robot's head through path integration using feed-forward commands and visual landmark detection. Compared to previous work on neuronal path integration, this work scales up the system to a higher resolution of pose representation, applies it to a 2D system of the robot's head, and quantitatively assesses the path integration performance.

We realize this model directly and fully in neuromorphic hardware—Intel's research chip, Loihi (Davies et al., 2018). We explore the model's function with a humanoid robot, the iCub (Metta et al., 2008), in the system designed to enable closed-loop experiments, i.e., the network controlling the robot's movement. The network tracks the movement in two degrees of freedom of the robot's neck. In our experiments, the iCub explores a wall with an object (a dotted pattern) on it by moving its head. Here, we do not use proprioceptive sensors (motor encoders or IMU) to estimate the robot's pose in an SNN; we use only the issued motor commands. This is done because we would like to estimate the precision of path integration in an SNN without mixing it with sensor errors in pose measurement. Moreover, sensors directly measuring the state of a joint are often not available in more complex motor systems or are costly (e.g., force sensors of compliant actuators). Such sensors can always be used to improve state estimation, similar to how vision is used in our model.

We use an event-based camera and simple visual preprocessing to estimate the position of an object in the field of view. More sophisticated event-based feature extraction could be used instead (Alzugaray and Chli, 2018; Gallego et al., 2019), but the visual processing was not our focus. When the object falls in the center of the visual field for the first time, the network stores the current pose of the robot's head, estimated in the network. Each time the object is seen in the center again, the stored pose is activated and used to correct the current pose estimate. The stored pose can also be used as long-term memory for object location and can trigger a goal-directed movement toward the memorized object, even if it is not in view.

The paper proceeds with a description of the hardware setup and the hardware and algorithmic interfaces between the robot and the neuromorphic chip. We then explain the SNN model and show results for pose estimation through path integration on-chip and vision-driven object-directed pose learning. We evaluate network performance in terms of the precision of state estimation and discuss how the SNN parameters influence it. Finally, we conclude with a discussion and the positioning of this work in state-of-the-art neuromorphic robotics.

## 2. Hardware Systems

### 2.1. The iCub Humanoid Robot

**Figure 1:**
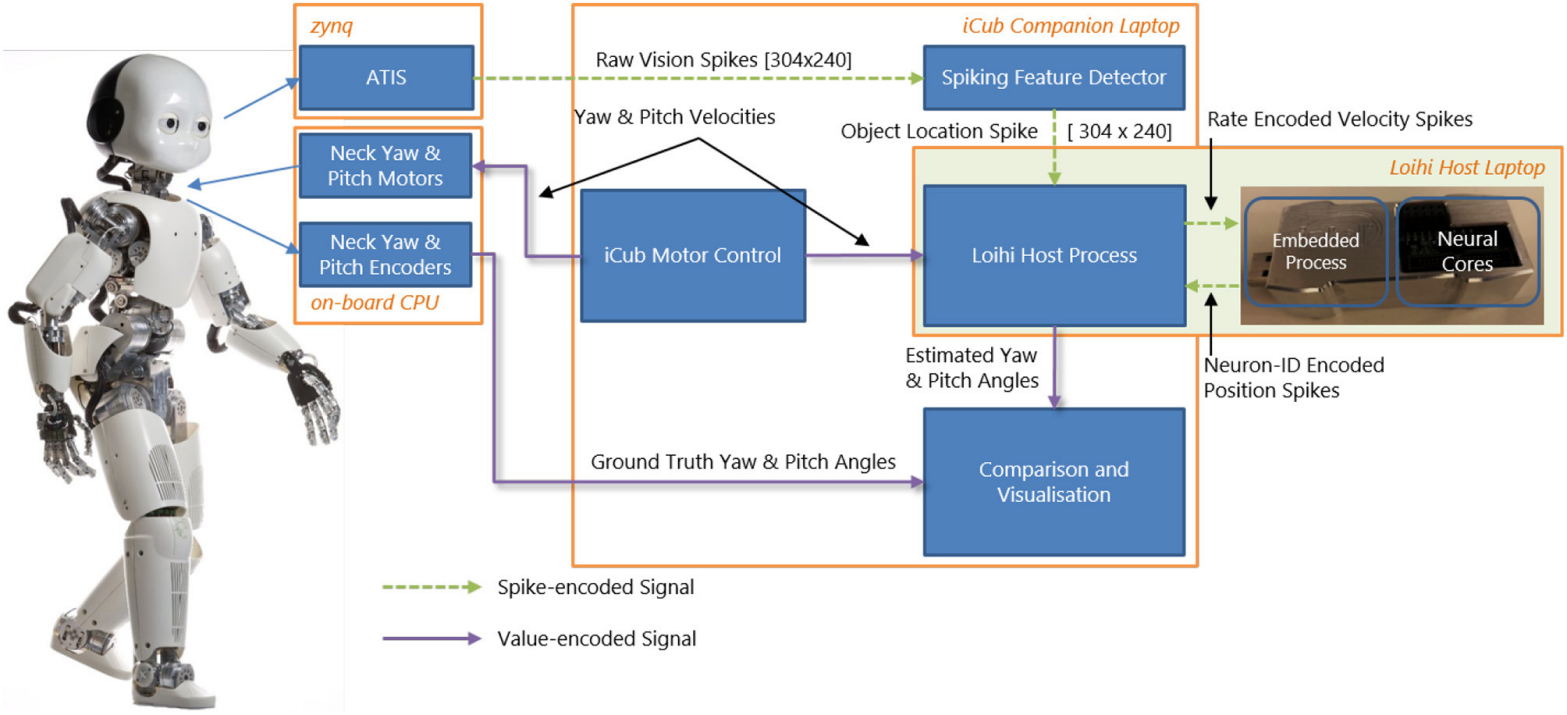
System overview of the integration of the iCub robot and Intel's neuromorphic research chip, Loihi, highlighting visual and motor components as well as spiking and non-spiking signals.

Our goal (beyond this paper) is to perform closed-loop experiments between the SNN and iCub. Therefore, we built an online interface between the humanoid robot iCub (Metta et al., 2008) and the neuromorphic device Kapoho Bay, which contains Intel's neuromorphic research chip, Loihi (Davies et al., 2018). An overview of the system is shown in Figure 1. We used YARP (Metta et al., 2006)—a middleware that allows seamless communication between different software components across the network—for modular processing and transparency between different computers and devices.

The neuromorphic iCub (Bartolozzi et al., 2011) has two event-based cameras, specifically, the Asynchronous Time-based Image Sensor (ATIS) (Posch et al., 2008), as part of its biologically-inspired vision system. The camera pixels produce asynchronous events as output. Each pixel emits an event when the level of sensed brightness changes by a certain amount. We used an event-driven visual tracking algorithm that produced “spikes” (event addresses) representing the target object position. This output was sent to the SNN on Loihi. Specifically, the visual input network in our model received input whenever the target object was in the field of view (as described in section 3.2).

The iCub's motors are controlled by sending velocity commands from a motor control loop. In our experiments, we moved the iCub head within two degrees of freedom, setting velocities for its yaw and pitch joints. We used movements at various speeds between the joint limits of the robot. The motor control module produced the following behaviors used in our experiments: a constant velocity along a single axis and a random-walk motion along both axes.

The iCub has encoders that count motor rotations for the six degrees of freedom of the head and neck. The encoders are usually calibrated by hand by initializing the robot in the 0° positions and measuring the encoder offsets to these positions. These offsets need to be updated whenever there is a mechanical change or after time due to wear. In this work, we did not use the position of the head read by the encoders to control the robot. Instead, we only controlled the motors' velocities along each axis directly, without sensory feedback on the motor position. Thus, the controller did not rely on the external calibration of the encoders. The head pose was estimated in the spiking head-direction network on the neuromorphic chip (section 4). The encoders were used solely to obtain the ground truth for experimental analysis and therefore provided no input to the algorithm.

The overall experimental system consisted of two laptops and the iCub robot connected to the same local network. We used the iCub middleware YARP (Metta et al., 2006) to connect different modules. For clarity, we briefly describe the exact computer configuration used. An iCub-companion laptop was used to run the motor control, which communicated with the iCub's on-board PC to move the robot. The motor commands (velocities) were at the same time sent over the network to the Loihi host laptop. The Kapoho Bay Loihi device was directly connected to this laptop by USB. The iCub companion laptop also read the raw camera events and ran the event-driven object detection algorithm. The object location spikes were sent to the Loihi host laptop. The output of the head-direction network was sent from the Loihi host laptop to the iCub companion laptop for visualization and recording; the encoder values were sent from the iCub on-board PC to the same module. During experiments, all signals were recorded except for the direct USB communication with the Loihi and the direct motor control with the iCub (the velocities sent to the Loihi host laptop were recorded instead).

Currently, to get these two cutting-edge, complex technologies to work together, the systems interface also has to be complex. One important contribution we make is to highlight this fact, with the aim of understanding how, in the future, we can develop a fully neuromorphic-integrated robot with fully spiking communications. We believe the system as we present it is still the required first step to doing so.

### 2.2. The Loihi Neuromorphic Research Chip

Intel Neuromorphic Computing Lab designed the neuromorphic research chip, Loihi, in which spiking neural network models can be simulated in real time efficiently (Davies et al., 2018). The chip consists of a mesh of 128 neuromorphic cores and three embedded ×86 processor cores. For this work, we used Kapoho Bay, the USB form factor version, which contains two Loihi chips. The chips are configured using a Python API provided by the Intel Neuromorphic Computing Lab (NxSDK 0.9) that allows us to define the spiking neural network on the level of groups of neurons and synapses. Loihi implements the leaky-integrate-and-fire neuron model as described by Davies et al. (2018) and allows flexible on-chip learning.

### 2.3. Hardware Interface Between iCub and Loihi Using YARP

Figure 1 gives an overview of the interfaces between the iCub robot and the Loihi neuromorphic research chip. From the robot, a copy of the movement commands (section 3.1) and the visual information (section 3.2) are sent via YARP to the Loihi host computer. On the latter, a program is running that sends the values of the motor commands and the visual spikes to the embedded process running on the ×86 processor on the Kapoho Bay device. The embedded process sends spikes received from the host to the neural cores and reads the spikes from the previous time step to send them out to the host process.

At the beginning of each trial, the embedded process waits for an initialization input from the host computer to confirm that the robot or data player is sending events. While the experiment is running, the host process is synchronized to the embedded process and the neural cores so that they all advance their algorithmic time steps at the same time. On average, one algorithmic time step takes about 1.6 ms. Most of this time is taken up by the output being sent from the embedded processor to the host computer to monitor the spikes. Section 3.1 discusses the time step duration issues. The output spikes of the head-direction network—from the “goal head direction” layer—are designed to be sent through a YARP port, to eventually control the robot's gaze to one of the stored object locations in a closed-loop experiment; however, this is planned for future work.

## 3. Algorithmic Interfaces Between Robot and Neuromorphic Chip

In this section, we describe how we generate input to the SNN on-chip model from motor commands and camera events and how we read out SNN activity.

### 3.1. Input Spike Generation Based on Velocity Commands

When the robot moves its head, the velocity commands are sent to both the robot and the host computer of the Loihi chip. On the host computer, a small C++ program receives the velocity commands that are interpreted as the neuron's input current *I*_*in*_ (in °/*s*) after being multiplied by the measured timestep duration on Loihi. Four of Loihi's integrate-and-fire neurons are dedicated to integrating the velocity input for yaw (left and right) and pitch (up and down) movements. A change in velocity, therefore, leads to an immediate change in input current and, with that, changes the neuron's membrane potential *V*(*t*), Equation (1).

(1) $$\begin{array}{l} \Delta V(t)=I_{in}\cdot \Delta t-V_{thr}\cdot \Theta (V(t)-V_{thr}),\text{ where}\\ \Theta (x)=0,\text{ if }x\leq 0;\\ \Theta (x)=1,\text{ if }x>0.\; \end{array}$$

Here, at every timestep, *t*, the current speed command *I*_*in*_ (in °/*s*) is multiplied by the measured duration of the timestep and added to the neuron's membrane potential *V*(*t*). Note that timesteps may take a variable amount of time in the system depending on spiking rates and other computational overhead. When *V*(*t*) of the velocity neurons surpasses a threshold value *V*_*thr*_, the neuron emits a spike, and the magnitude of *V*_*thr*_ is subtracted from the membrane potential to reset the neuron. The *firing rate* of the velocity neuron is thus proportional to the velocity command sent by the motor controller of the robot, and the proportionality coefficient can be controlled by the threshold parameter *V*_*thr*_. The emitted spikes then stimulate the shift layer of the SNN model, as explained in section 4.

The value of *V*(*t*) is clipped at a maximum value of *V*(*t*) = 2*V*_*thr*_. Furthermore, we added a refractory period that prevents the input neuron from firing more often than every third timestep, which is the time that the head-direction network needs to fully integrate an input spike in our “every spike matters” setting.

The threshold *V*_*thr*_ of the simulated velocity input neurons determines the quantization step and the path integration rate of the network. For instance, if we set *V*_*thr*_ = 0.5°, the velocity neuron will produce a spike whenever the robot has moved its head by 0.5°. This spike shifts the current estimate of the head angle in the head-direction network's activity by one neuron within *n* = 3 time steps, so we need 200 neurons to represent an angle of 100°. Assuming an average timestep duration of 1.6 ms, we can calculate that if we set the threshold to *V*_*thr*_ = 0.5°, the SNN activity can faithfully follow an angular velocity of approximately $\omega =\frac{v_{thr}}{n\Delta t}\approx 100^{◦}{\text{s}}^{-1}$. This sets the maximal speed at which the activity can be shifted in our SNN model.

**Figure 2:**
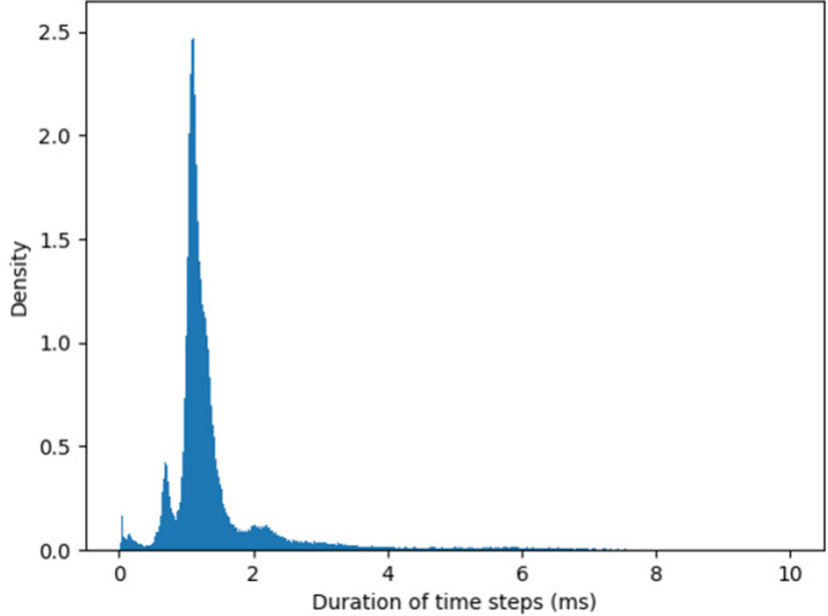
Histogram of the algorithmic time step duration as recorded by YARP in our experiments. The average timestep is 1.6 ms, but in rare cases, time steps can be as long as 40 ms. Note, these values hold for the specific version of the Loihi API used.

The timestep can be further shortened (and the maximal velocity increased) by optimizing the I/O from the chip. The duration of timesteps fluctuates as SNN simulation unfolds in real time. Figure 2 shows the distribution of the measured timestep duration in our experiments.

Note that all other neurons besides the velocity input neurons receive spikes from connected neurons as input instead of a direct change in current. The input to these neurons is the sum of filtered spikes from connected neurons, leading to a synaptic response current *I*_*in*_(*t*) (Davies et al., 2018). By default, after each spike, the membrane potential *V*(*t*) is reset to zero instead of subtracting the threshold. Although this neuron model is often used as an input integrator in computational neuroscience, it might lead to “loss” of input current at large inputs and consequently to an error at the value-to-spikes interface. We thus introduced the “soft-reset” in the input layer to achieve maximum accuracy of pose representation in the network. At timepoints with a reliable visual reset or when other external sensing can be used to correct path integration, this input-integration error can be neglected.

### 3.2. Spiking Object Detector

The fundamental purpose of the vision system is to give a consistent signal about head pose that is not affected by integration drift. The signal is not explicitly known *a-priori*, i.e., we don't know where an object will be, but given any pose, the visual signal will not change over the course of the experiment. However, the relationship between pose and visual signal still needs to be learned during the experiment.

Our eventual goal would be to use an event-driven object detection system (e.g., Liang et al., 2019) as an SNN inside the Loihi chip. However, in this paper, we are focusing on the path integration and visual reset in the SNN rather than a complex visual system. One other current integration issue is the data bandwidth to the Kapoho Bay, which limits the amount of visual data that can be sent to the chip. Therefore, for this paper, we used a simplified spiking object detector outside the SNN (in software) based on a visual tracking algorithm to encode the position of a single object in the visual field of view. In our current setup, we consider only a single object in the visual field of view. However, more complex recognition systems can be extended to multiple objects.

The spiking object detector receives the raw events from all pixels of the ATIS neuromorphic camera (Posch et al., 2008) and outputs spikes associated with the object position, *O*_*xy*_. The output array of the detection, therefore, has the same dimensions as the sensor itself, 304 × 240 pixels. The starting position of the object of interest $O_{xy}^{0}$ was marked in an initialization phase in which the user sets the correct position of the object. Following initialization, tracking was achieved by setting a region of interest of size *R*_*size*_ around the initialized point. When *N*_*events*_ camera events were received in the region of interest, the mean position of the events was calculated, and the output neuron produced a spike at the position of the object in the visual field, $O_{xy}^{t}$. The new region of interest was defined around the updated mean-firing position, $O_{xy}^{t}$, and the events were again accumulated within the region of interest in order to produce the subsequent spike.

The output of the object detector is event-based: its firing rate depends on the rate of camera events within the region of interest. A single spike is output at the moment in time that the object position moves by 1 pixel. The resolution of the temporal precision of the output is under 1 ms.

The detector's output spike is sent to the spike-generator interface on the Loihi host computer and sent to the neuronal cores, to the visual input network. The visual input network receives the detector spikes according to their position in the visual field with a rectangular 2 × 2 pixels receptive field. The central neuron of this array activates the visual reset neuron.

### 3.3. Reading Out the Head Direction From the Network

At every timestep, a data package containing the indices of the currently firing neurons (“address event representation”) is sent by the Loihi embedded process to the Loihi host computer. Since the total processing time is dominated by sending the output packages to the host computer, we only record the neuronal populations required for the system's performance evaluation. In our place-code representation, the spike's index (“address”) directly corresponds to the represented variable value, e.g., the yaw or pitch.

## 4. The Head-Direction SNN

### 4.1. Network Overview

**Figure 3:**
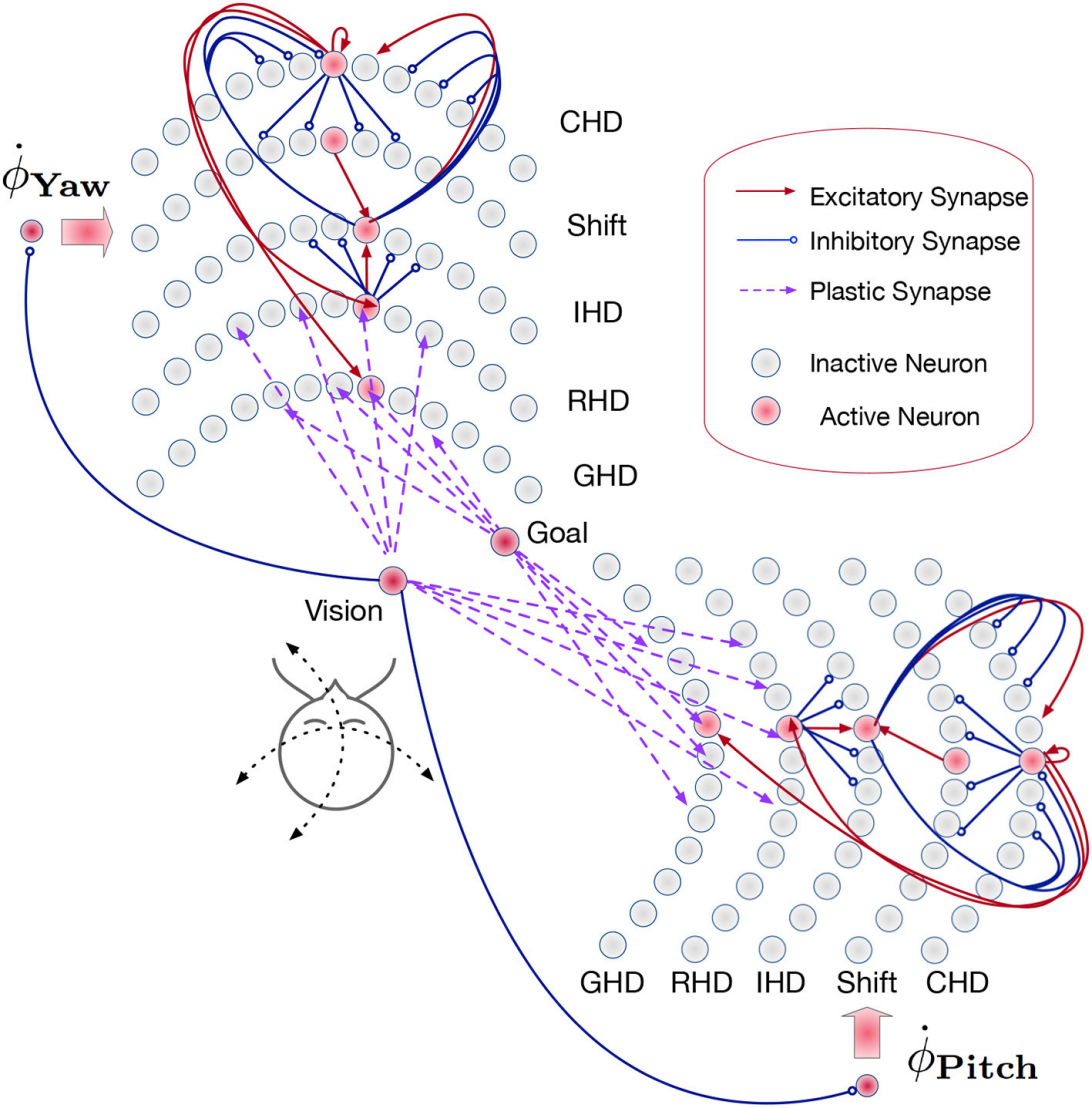
Overview of the path integration and map learning network: Two identical SNNs with five functional layers each estimate the yaw and pitch of the iCub's head pose, integrating the respective motor velocities. When the robot's gaze is directed at a visual landmark, the yaw and pitch angles are stored in plastic synapses connecting the Vision and Goal neurons to the Reset Head Direction and Goal Head Direction layers, respectively. Excitatory synapses (red lines) between layers connect neurons in a one-to-one manner. The velocity input neurons are connected to shift layers in a one-to-all manner. Plastic connections (purple lines) are one-to-all, and inhibitory connections (blue lines) are all-to-all-but-one. Only exemplary connections are shown, in order to avoid clutter. See section 4.3 for details.

The path integration network consists of two identical SNNs for yaw and pitch estimation (Figure 3). Each of these SNNs, similar to networks used in Kreiser et al. (2018a,c), consists of six layers of *N* = 200 neurons each:

- the current head direction layer (CHD),
- the shift left layer (SL),
- the shift right layer (SR),
- the integrated head direction layer (IHD),
- the reset head direction layer (RHD), and
- the goal head direction layer (GHD).

The input to each of the two networks comes from two velocity populations [one for clockwise (“right”) and one for counter-clockwise (“left”) movement] and several visual-landmark (visual-reset) populations. We can have as many of these populations as there are landmarks or objects known to the robot. As Loihi is a digital, deterministic neuromorphic system that does not require redundancy to cope with mismatch and noise in neuronal dynamics, each of the velocity input and visual landmark populations consists of a single neuron in our implementation.

### 4.2. Functional Description of the Network

In the *Current head direction (CHD) layer* of the yaw and pitch path integration SNNs, the current pose (yaw or pitch, respectively) of the robot's head is encoded in the position of the active neuron: each neuron corresponds to a specific value of yaw or pitch. We thus use one-hot encoding. At the start of every trial, the neuron that codes for the initial position (the central neuron) is activated. An active neuron in the CHD layer inhibits all but one neuron in all Shift layers: only neurons with the same index as the active CHD neuron can be activated.

The *Shift layers* are responsible for shifting the position of the active neuron to the left (Shift Left, SL) or the right (Shift Right, SR). An entire shift layer is activated by the respective velocity neuron through a “boosting” one-to-all connectivity pattern. The SL layer is activated by a counter-clockwise movement command and the SR layer by the clockwise movement command. During visual reset, the shift layers are inhibited by the visual landmark population. Both shift layers project their activation to the IHD layer with a one-to-one-shifted connectivity pattern. The IHD layer integrates this “shifted” CHD activation with the visually-driven reset input.

The *Reset head direction (RHD) layer* is active when it receives input from the visual-landmark population, which arrives through plastic connections that are learned when the landmark is seen in the center of the visual field for the first time. The plastic weights store the pose (head direction angle: yaw or pitch) that the robot had when it was looking at the landmark for the first time. When the landmark is revisited, the strong potentiated plastic synapses drive an activity bump in the RHD layer. Weak input from CHD is not sufficient to induce activity in the RHD on its own. If the RHD layer is active, it resets the activity in the IHD layer through a set of strong weights: an active RHD neuron excites the corresponding neuron in the IHD layer and inhibits all other neurons (the “reset” connectivity pattern).

The *Goal head direction (GHD) layer* behaves exactly the same as the RHD layer but is only a readout population with no outgoing connections to the other parts of the network. It is used in a scenario of goal-directed behavior to look at the learned object. It receives the same subthreshold activation from the IHD layer and additionally receives input through plastic synapses from a goal population that is activated by the visual landmark input. As in the RHD layer, the plastic weights leading to the GHD layer act as a memory that associates a specific landmark with a pose.

Finally, *Integrated head direction (IHD) neurons* project in a one-to-one manner to the CHD layer, also with inhibition to all other CHD neurons (“reset” pattern), thus either shifting the activity location if no visual landmark is detected or resetting this activity to an updated location if a visual landmark dictates such an update.

### 4.3. Connectivity in the Head-Direction SNN

To achieve the described behavior, the layers of the model are connected as shown in Figure 3. Note that all weight values in the description below and the parameter tables are given in multiples of the neuron threshold (*V*_*thr*_ = 100 here).

In the CHD layer, every neuron excites itself with a weight of *w*_*CHD*_*CHD*_ = 1.2 so that the activity of the network is self-sustained. I.e., the current pose is stored until it is set to a different position by the IHD.

The CHD layer is connected to the shift layers with all-to-all-but-one inhibition (“negative preshape” connectivity pattern) so that only the corresponding neuron can be activated by the one-to-all excitatory input from the velocity populations (“boost”). The shift layers have shifted one-to-one synapses to the IHD.

RHD and GHD receive a subthreshold (*w*_*CHD*_*RHD*_ = *w*_*CHD*_*GHD*_ = 0.2) one-to-one input from the CHD layer that is used to learn the initial pose of the landmark (“preshape” connectivity pattern).

Plastic one-to-all connections from the visual-landmark population add input to the RHD neurons and drive the neuron with a pre-shaping CHD input above the threshold. The plastic connections between this neuron and the visual landmark neuron are updated then and store the pose of the iCub's head that corresponds to having the landmark in the center of the visual field. After learning, plastic connections form a one-to-one connectivity pattern: a single synapse from the visual neuron to the correct RHD neuron is potentiated (high); all other synapses are depressed (low).

A goal neuron is connected via one-to-all plastic synapses to the GHD layer. The goal neuron can be driven externally to remember the pose-landmark association without resetting the current pose estimate through the IHD layer. The goal neuron receives excitatory one-to-one connections from the visual landmark neuron to learn the pose-landmark associations.

The RHD layer has one-to-one excitatory and all-to-all-but-one inhibitory connectivity (the “reset” pattern) to the IHD layer to override input from the shift layers when the visual reset is active. The IHD layer is connected to the CHD layer with all-to-all-but-one inhibition (*w*_*IHD*_*CHD*_*inh*_ = −1) to delete the previous state and one-to-one excitation (*w*_*IHD*_*CHD*_*exc*_ = 1.24) to “copy” the current state.

The learning rule of the plastic synapses between the visual-landmark neurons and the RHD/GHD layer is specified as follows:

(2) $$\Delta w=y_{0}\cdot x_{1}-\text{λ}\cdot x_{0},\text{ if }w<w_{\max}.$$

Here, Δ*w* is the weight update at a given timestep. *x*_0_ ∈ {0, 1} and *y*_0_ ∈ {0, 1} are variables that become 1 if there is a pre- or post-synaptic spike, respectively. *x*_1_ is a variable that stores an eligibility trace of the pre-synaptic neuron activity, and it decays over *n* time steps (*n* = 2 here, since the post-synaptic spike should arrive in the next time step); *w*_max_ = 256 is a maximal weight value at which weights saturate.

According to the learning rule (Equation 2), a synapse potentiates if an RHD neuron (post-synaptic) fires after the visual-landmark neuron (pre-synaptic) fired. The closer in time the visual-landmark neuron fires to the RHD neuron, the higher the pre-synaptic trace *x*_1_, leading to a more significant weight update. Synapses are depressed (decrease) by a constant factor of λ if the post-synaptic (RHD) neuron did not fire but the pre-synaptic neuron (visual) did fire. The weights are initialized to a subthreshold value (*w* = 0.8) so that, together with the input from the CHD, their summed input activates the RHD at the currently estimated pose. This leads to one-shot learning of the pose, while all other synapses that connect to non-active RHD neurons are depressed to 0. Plastic synapses between the goal and GHD neurons are learned in an online fashion throughout the whole experiment: learning was not artificially stopped at any time.

Table 1 lists all neuronal and synaptic weight parameters used in the head-direction SNN and their values.

**Table 1:** Values of synaptic weights between layers in the head-direction SNN on Loihi and parameters of neurons.

**Parameter**	**Value**	**Parameter**	**Value**	**Parameter**	**Value**
*w*_*CHD*_*CHD*_	1.2	*w*_*CHD*_*Shift*_	−0.5	*w*_*Velocity*_*Shift*_	1.0
*w*_*Shift*_*IHD*_	1.0	*w*_*IHD*_*CHD*_*exc*_	1.2	*w*_*IHD*_*CHD*_*inh*_	−1.0
*w*_*CHD*_*RHD*_	0.2	*w*_*RHD*_*IHD*_*exc*_	1.0	*w*_*RHD*_*IHD*_*inh*_	−0.7
*w*_*vision*_*RHD*_*initial*_	0.8	*V*_*thr*_	100	τ_*V*_, τ_*i*_	1

*All weights are given as multiples of V_thr_ (i.e., they are multiplied by 100 before being set on the chip). Both time constants of neurons (τ_V_) and synaptic temporal filters (τ_i_) are set to 1 timestep, which means that each neuron's membrane potential is reset after every timestep, i.e., neurons do not keep a state. Memory in the network is maintained using the recurrent connectivity*.

## 5. Experiments and Results

We describe experiments in which the proposed SNN model estimates the head pose of the iCub robot. Three evaluations were performed:

- We assessed the accuracy of the integration component of the head-direction SNN without visual input.
- We assessed the improvement of the network with visual learning and reset.
- We investigated the representation of the object location in the SNN and how it relates to the map creation.

### 5.1. Experimental Setup and Dataset

Data for repeatable experiments were produced using the *neuromorphic iCub* robot (Bartolozzi et al., 2011). We evaluated the entire system using online, live experiments connecting the SNN and robot. However, the results presented were produced on recorded data to ensure reproducibility. The datasets are available permanently and can be downloaded here^1^. There are five datasets with random head movements and a simpler one with a squared movement of the head. Each dataset contains ATIS features, visual tracker output, motor commands, and robot encoder values. All datasets start with a calibration phase (0–25 s) where the robot performs independent yaw and pitch movements.

The yaw and pitch motors of the iCub head were controlled to maintain the desired velocity during operation. By using only velocity commands, the head-control module has no information about the position of the joints, and in the main part of the architecture, the only module that estimated the pose was the head-direction SNN. We used the encoder values in auxiliary functions to enforce joint limits (to avoid robot damage) and to center the head between trials, which was required for repeatable experiments. Encoder values were also used as a ground truth against which we compared the activity of the head-direction network. All parameter values used in experiments are listed in Table 2.

**Table 2:** Parameters of iCub movements in the experiments.

**Parameter**	**Value**	**Parameter**	**Value**
$j_{min}^{0}$	−20 deg.	$j_{min}^{2}$	−35 deg.
$j_{max}^{0}$	10 deg.	$j_{max}^{2}$	35 deg.
$v_{1}^{0}$	7 deg./s	$v_{1}^{2}$	14 deg./s
$v_{2}^{0}$	14 deg./s	$v_{2}^{2}$	28 deg./s
$v_{4}^{0}$	28 deg./s	$v_{4}^{2}$	56 deg./s
*r*_*timeout*_	3 s		
*R*_*size*_	50 pix.	*N*_*events*_	1000

The iCub robot performed the following behaviors:

- **Home:** The head is moved to the center of the workspace, controlling the velocity, such that new trials begin with identical pose.
- **Nodding:** The robot nods its head upward and downward between the joint limits ($j_{min}^{0}$ and $j_{max}^{0}$). No horizontal motion.
- **Head-shaking:** The robot shakes its head side-to-side between the joint limits ($j_{min}^{2}$ and $j_{max}^{2}$). No vertical motion.
- **Random:** The robot chooses random velocities at which to move both vertically and horizontally. The velocity is chosen as a uniform distribution between 0 to *v*^0^ and 0 to *v*^2^ for the vertical and horizontal motion, respectively. If a joint limit is reached, the velocity of the respective joint is reversed. New velocities are chosen after *r*_*timeout*_ seconds.

In addition to direction, we changed the speed of the robot's movements: $v_{1}^{0}$ and $v_{1}^{2}$ are the base velocities used, and during experiments, the speed was increased such that *v*_2_ is double and *v*_4_ is four times the base speed, applied to both joints simultaneously (see Table 2).

Five datasets were recorded with the robot beginning in the home position and then proceeding with the following strategy: nodding, home, head-shaking, home, random with speed *v*_1_ for ~30 s, random with speed *v*_2_ for ~30 s, random with speed *v*_4_ for ~30 s, and finally, home. The data were recorded from one of the ATIS cameras on the robot after a pre-processing stage to eliminate the saving of uninformative events (a noise filter). The motor-control module output the velocity of the head when the commanded velocity changed; the data were saved along with the iCub head encoder values. All data were timestamped to synchronize during playback correctly and for further processing.

The data were saved and processed offline to enable a repeatable analysis of the visual integration; however, the entire pipeline was also tested with the robot in the control loop. Therefore the system is capable of estimating the robot's head pose and memorizing object-directed poses in real time during the robot operation.

**Figure 4:**
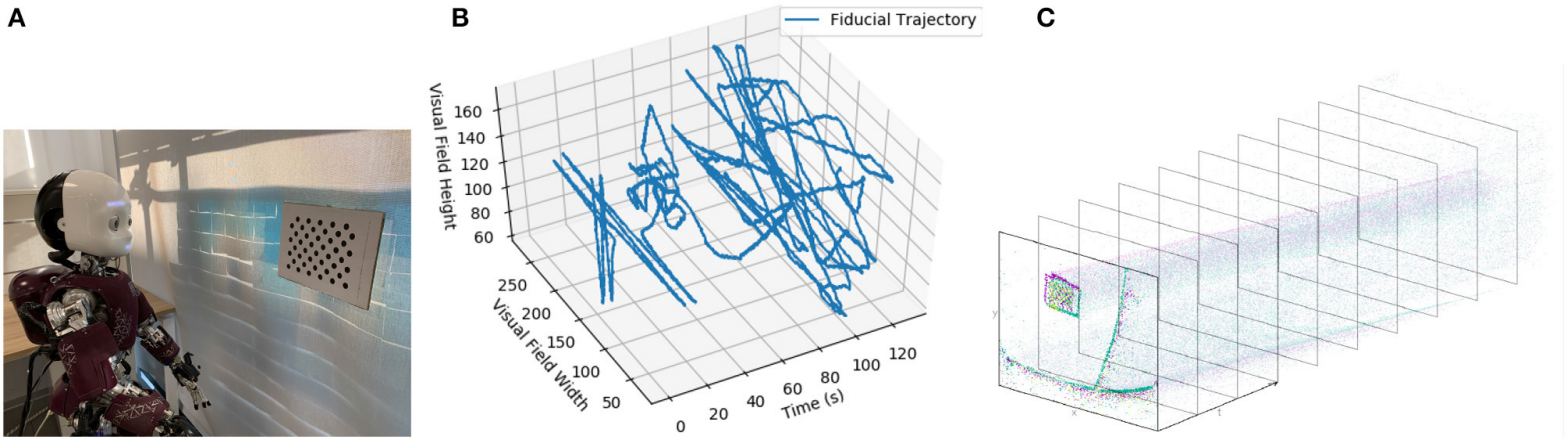
**(A)** The iCub robot and visual fiducial (dot pattern). **(B)** The visual trajectory of the fiducial over dataset 1. **(C)** An example of the ATIS camera output.

The robot was positioned to look at a dot pattern, which was chosen because it produced a strong signal in the visual stream (Figures 4A,B). The background of the scene was predominantly a blank wall to avoid the interference that would be introduced by a cluttered scene (as visual processing was not the focus of this experiment) but also included desks and windows. The dot-pattern was placed in different positions relative to the robot for each of the five datasets. The position of the dot pattern in the visual array of the ATIS sensor was extracted from the visual stream by visual tracking (Figure 4C), section 3.2. Both the position of the dot-pattern and the commanded velocities of the robot's head motors were sent to the Loihi host process to be converted to spikes compatible with the SNN on the Loihi neural core. The SNN produced the estimate of the iCub's head position, which was recorded and compared to the encoder values and path integration in software.

### 5.2. Integration-Only Pose Estimation

**Figure 5:**
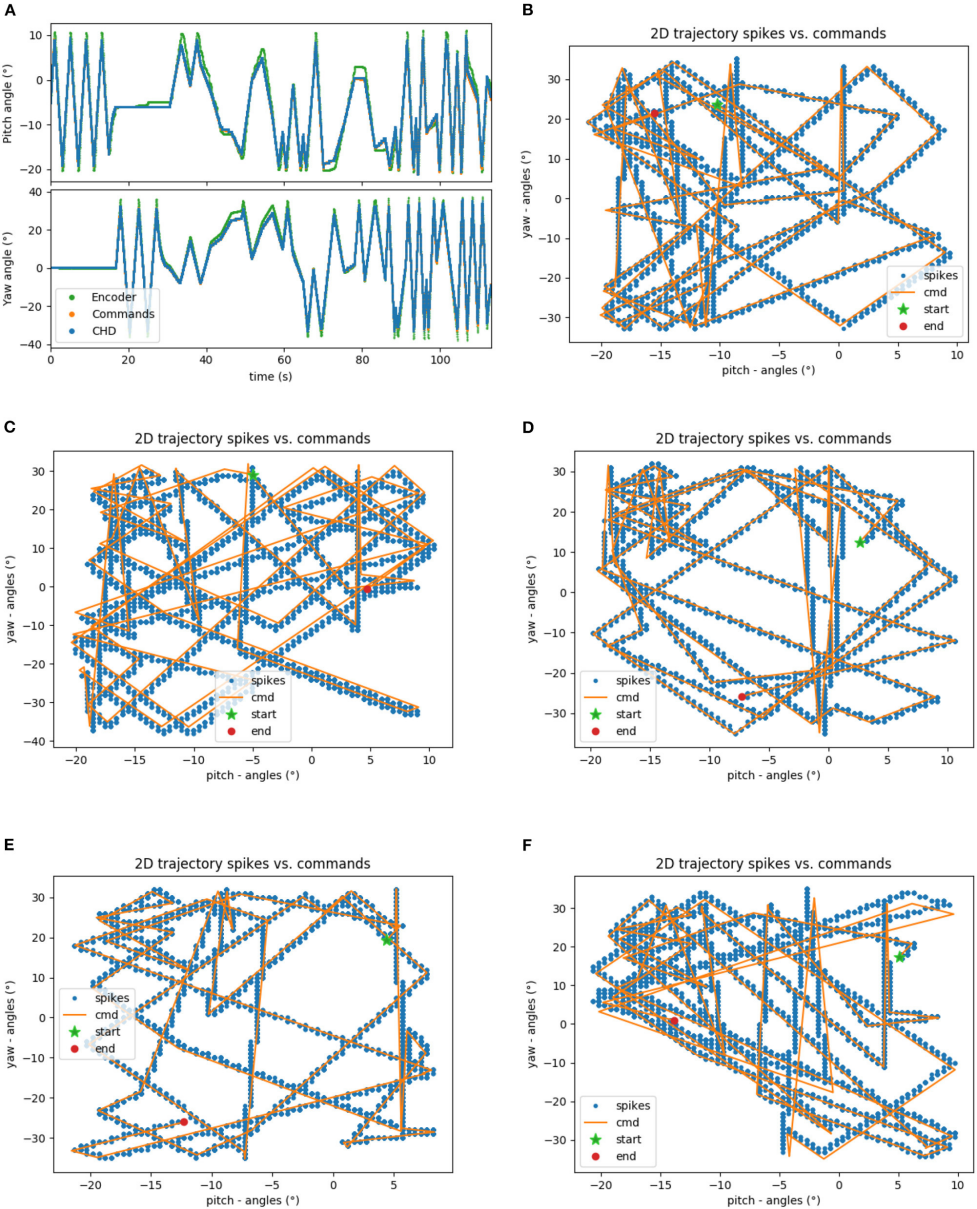
Results of robotic experiments on path integration in head-direction SNN on chip. **(A)** The estimated yaw and pitch angles over time for dataset 5. A match between the SNN-estimated pose and integrated motor commands can be observed, with small deviations from actual movement as measured by the motor encoder. **(B)** The same trajectory in 2D motor space. **(C–F)** Trajectories for the datasets 1, 2, 3, and 4, respectively.

The head-direction SNN was initially evaluated on its ability to integrate the velocity commands to estimate the position, without any correction from the visual system. The robot began each trial in the center of the workspace. The head-direction network was initialized with an active neuron in the center of the CHD layer. With the correct input threshold (calculated as described in section 3.1) applied to the integration dynamics of the network, we achieved a close correspondence between the ground-truth pose calculated in software and yaw and pitch angles estimated by the SNN, as can be qualitatively seen in the time course of, e.g., experiment 1 shown in Figure 5A. Here, the yaw and pitch angles estimated in the SNN on-chip (blue line) and in software (orange line) overlap perfectly. Table 3 lists the RMSEs of dataset 5 using different thresholds *V*_*thr*_. We also show the value measured by the motor encoders (green line) for completeness. Trajectories in the 2D joint (yaw-pitch) space for all five datasets are shown in Figures 5B–F. Note that in the last example, the actual movement, measured by the motor encoders, deviates more strongly from the motor commands, which will be noticeable later in the learned map.

**Table 3:** Root mean squared errors (RMSE) in degrees for different thresholds of velocity input neurons, *V*_*thr*_, for the yaw and pitch estimation.

**RMSE (in ^◦^)**	***V*_*thr*_ = 2**	***V*_*thr*_ = 1**	***V*_*thr*_ = 0.5**	***V*_*thr*_ = 0.25**
Pitch	0.86	0.95	0.31	0.23
Yaw	1.54	2.04	0.58	0.58

*RMSEs are calculated based on time-aligned differences in angles estimated from the CHD layer of the SNN and calculated by path integration in software*.

Quantitatively, the RMSE between the estimated pose and pose measured by the encoders was 1.93° and 2.43° for the yaw and pitch angles, respectively, for our 2-min long experiments. Errors compared to the pose measured with motor encoders appear due to the inertia of the robot's movements. The encoders capture the actual position of the motors, and the joint motors are affected by inertia and other higher-order dynamics; i.e., the head cannot instantly change velocity. The head-direction network, to the contrary, immediately integrates the changing velocity—the motor commands are integrated precisely.

Further, we compared the estimated pose from the CHD layer to a software-based integration of the command signals performed with precise floating-point computation. To compute errors, we sampled the software pose estimation with a fixed interval of 2 ms and linearly interpolated to the spike times of the SNN-based pose estimation. The error was found to be 0.31 and 0.58° for the pitch and yaw angles, respectively. Table 3 lists the RMSEs of dataset 5, comparing the SNN estimation with command-based path integration in software for different settings of *V*_*thr*_. A *V*_*thr*_ = 2 corresponds to a network with *N* = 25 neurons in the CHD layer, *V*_*thr*_ = 1: *N* = 50, *V*_*thr*_ = 0.5: *N* = 100, and *V*_*thr*_ = 0.25: *N* = 200.

These encouraging results demonstrate the strong potential of the network as a head-direction estimator.

### 5.3. Visual Reset of Imprecise Pose Tracking

A correctly parametrized head-direction SNN is able to integrate the motor commands with high accuracy. However, when running for longer periods and with other (external) disturbances, it cannot be guaranteed that the estimate will always remain accurate. To simulate a disturbance within the shorter time frame of our recorded datasets, we artificially introduced a bias into the network. We show that the visual input and the visual reset layer of the network allow the pose estimation to be corrected.

**Figure 6:**
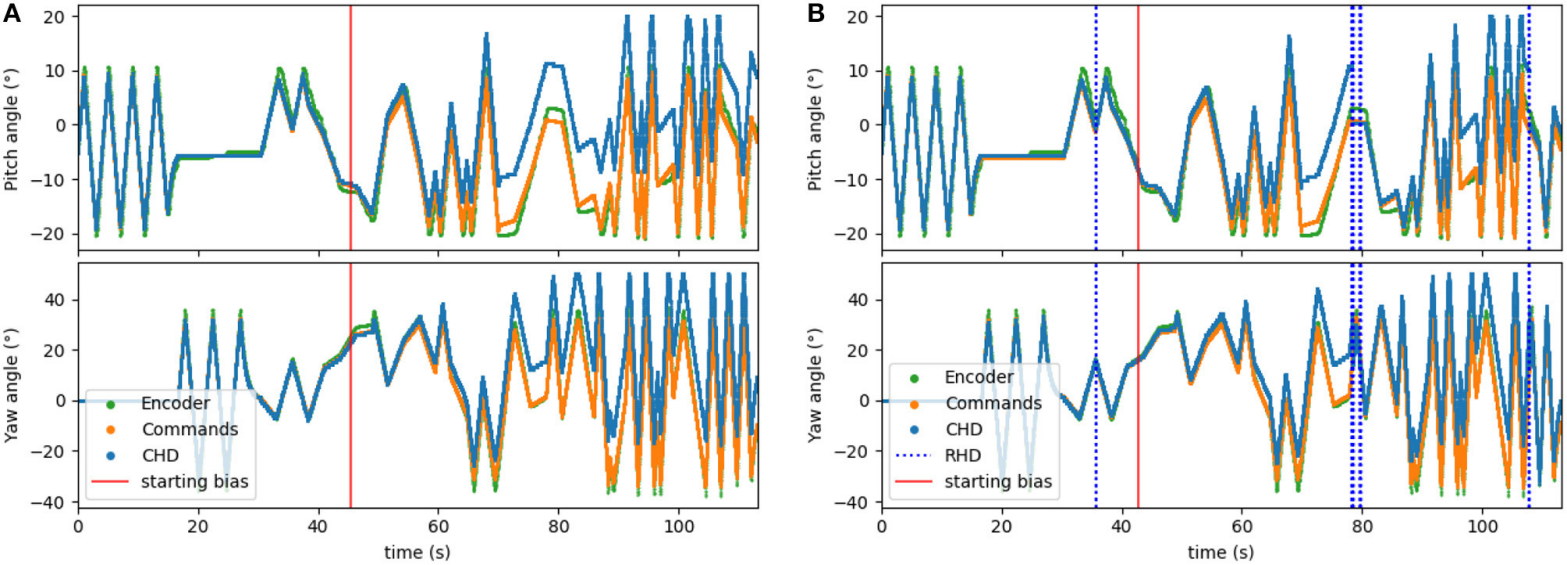
Testing the visual reset. After ~45 s (30,000 time-steps), the clockwise velocity signal is scaled by a factor of 1.1 as a simulated disturbance in the neuronal estimation of head direction. **(A)** Pose estimation without visual reset diverges from the ground truth. **(B)** Pose estimation with visual reset when the object is revisited. The dashed blue lines indicate the presence of the visual stimulus in the center of the visual field. The first blue line (around 36 s) indicates when the object was learned.

To corrupt the path integration, we multiplied the velocity signal, *I*_*in*_, in Equation (1) by a factor of 1.1 for the clockwise direction. We applied this bias after ~45 s (30,000 time steps) of the experiment. The performance of the resulting biased network can be seen in Figure 6A. Here, the yaw and pitch estimated in the CHD layer of the SNN (blue line) diverge from the software-integrated commands (green line) after the 45-s mark.

The visual reset component of the network allowed the estimated head direction to be corrected. In Figure 6B, the same biased network is used, but the active neuron in the CHD layer is reset when the target object is again seen in the center of the visual field. At the points of visual reset, the pose “jumps” to the pose learned when the robot looked at the object for the first time, thereby correcting the pose estimation. The RMSE, when compared to encoder information (see Table 4), is 6.05° in pitch and 11.10° in yaw for the corrupted network without visual reset and 4.47° in pitch and 8.98° in yaw when the detected landmark corrects the network during visual reset. The visual correction could potentially improve overall performance, removing the discrepancy between the motor commands and actual movements. However, our visual preprocessing itself was not precise enough to achieve improvement here.

**Table 4:** Root mean square errors (RMSEs) of pose estimation by the biased head-direction network with and without visual reset.

**RMSE, ^◦^**	**With visual reset**	**Without visual reset**
Pitch	4.47	6.05
Yaw	8.98	11.10

*RMSEs are calculated between SNN output and motor commands integrated in software*.

The visual reset component of the network is potentially more than just a correction tool. As we have shown previously (Kreiser et al., 2019a,b), this “loop closure” event can also be used to calibrate the gain of path integration, such that manual parametrization of the velocity input layer becomes unnecessary.

### 5.4. Representing the Visual Scene in the Network (Map Formation)

Although our simple visual pre-processing did not allow us to use multiple objects in the visual scene, the network can learn poses that correspond to looking at multiple objects. To demonstrate this, we concatenated five datasets with different object positions (two positions were the same). Each target was considered a unique object, and a new pose was learned for each object without forgetting the other ones. To achieve this, we introduced multiple visual landmark neurons. Each landmark neuron was activated by the object detected in the central part of the field of view. Here, we let different landmark neurons be activated in each of the five datasets. This manual neuron selection is a placeholder for the output of a fully-fledged object recognition system (e.g., Liang et al., 2019).

The network was successfully able to store multiple different objects with the plasticity mechanism described in section 4.3. We visualize the learned object-directed poses by activating each of five visual landmark neurons and reading out activity in the goal head direction (GHD) layer. The resulting 2D motor poses are shown in **Figure 8B** (colored crosses), compared to the ground truth of the encoder values read out when the robot was centering the target object in its field of view (colored squares).

**Figure 7:**
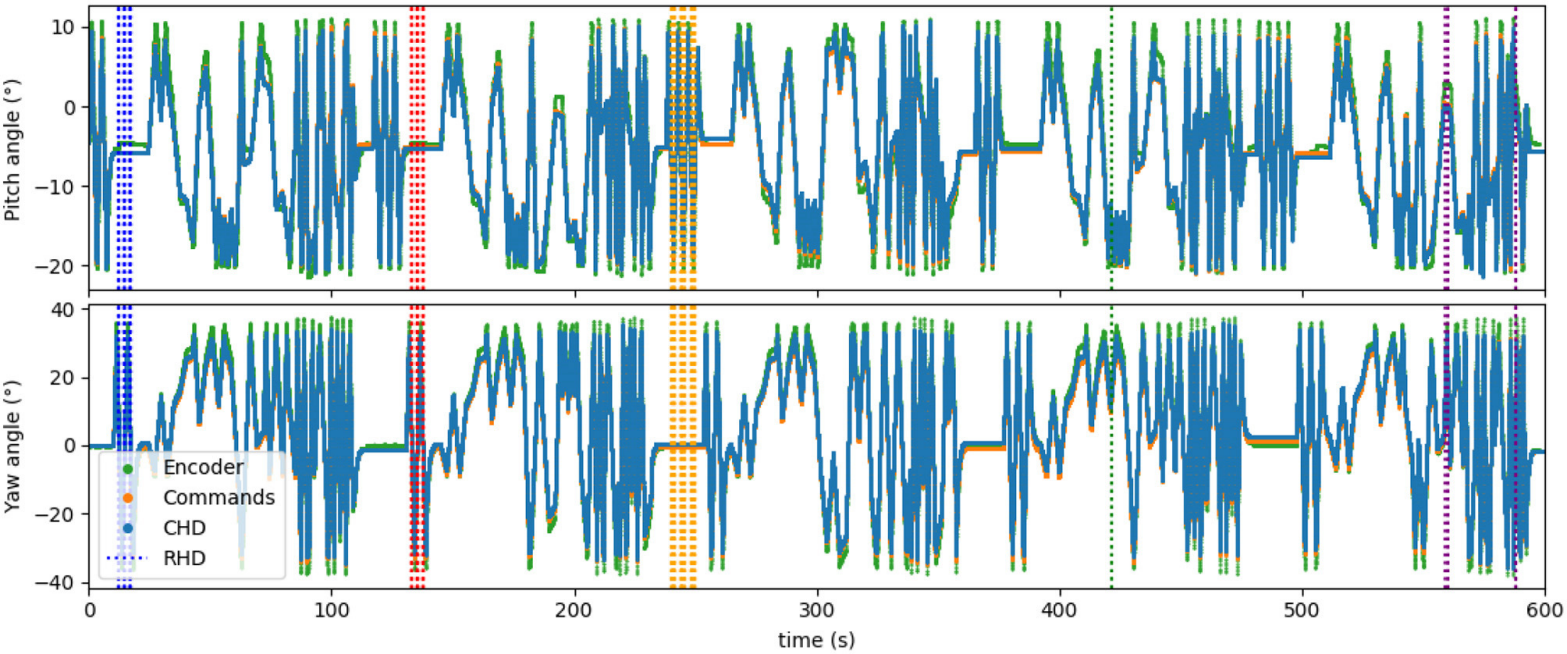
Five datasets concatenated in time, leading to about 10 min of recorded data. Visual learning and recall are indicated by the dashed vertical lines, with each color indicating a “new” object (object in a different location). Five different poses are stored in the network.

Figure 7 shows the time course of the whole experiment with five concatenated datasets. Visual resets occurred throughout the entire 10 min of dataset, periodically correcting the drift that accumulated over time. This demonstrates that visual reset is helpful even if the path integration is precise when the pose is tracked for a long time.

At the end of the experiment, the goal neurons that represent the five different landmarks were activated one by one, and the associated pose was recalled through the activity of neurons in the GHD layer.

**Figure 8:**
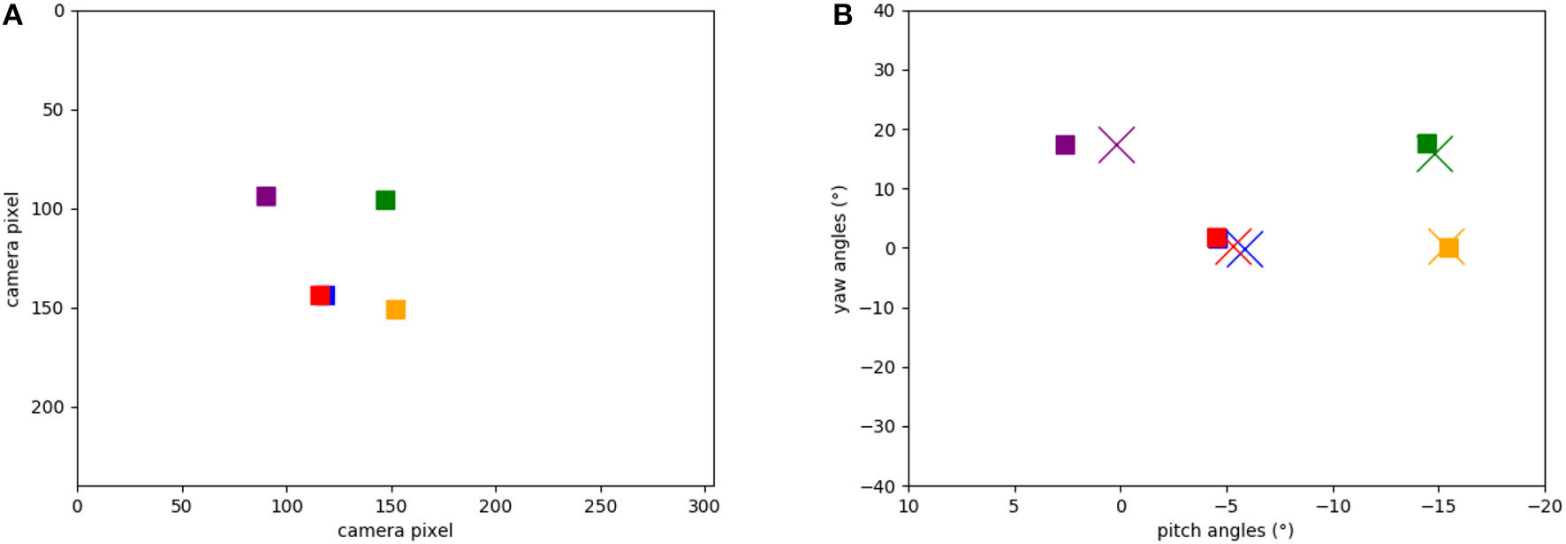
Positions of the objects in our experiments: **(A)** Positions of the objects calculated from ATIS events (in camera pixels when the robot looks straight). **(B)** Learned motor poses in the head-direction network (crosses) and object-directed poses calculated from the encoders (squares). Note that red and blue squares are on top of each other.

Figure 8A shows the five target locations in the camera's field of view, extracted from the events of the object tracker during the calibration phase when the robot's head is moved up and down and left to right. The location of each object was extracted from the intersection of event-traces during the yaw and pitch movements. Figure 8B shows the respective five positions in the robot's motor space: recorded from the encoders at the time when the landmark was in the center of the visual field (squares) and learned by the SNN (crosses). Each marker represents the pose that the robot needs to take to “look” at the respective object, i.e., center an object in its visual field. Note that there is a close match between the estimated pose and the ground truth (measured movement).

The learned landmark-centering poses can be used to direct the robot's gaze to the memorized object locations. E.g., the vector-integration to end-point (VITE) neuronal motor-control model generates movement based on the currently estimated pose and the stored goal pose (Grossberg, 1988). Alternatively, one can use a saccadic eye-movement-generating neuronal architecture (Bell et al., 2014; Sandamirskaya and Storck, 2014, 2015) to initiate the gaze to the memorized pose.

Note that during all of our experiments with recorded data, the data were fed to the SNN on-chip in real time, at a speed at which the real robot would provide the same data. Thus, no re-parametrization of the network was needed to run closed-loop experiments with the robot, and learning can proceed alongside the behavior in real time.

## 6. Discussion

In this work, we applied elements of neuromorphic SLAM—neuronal path integration, visual reset, and map learning—in the new setting of a humanoid robot observing a visual scene. The main results of this work can be summarized as follows:

- We have shown that even a small population of spiking neurons can perform precise path integration of motor commands to obtain an estimation of the current pose of the robot's head. The error, compared to path integration in software, accumulated over 120 s of the experiment, was at the resolution of value representation, < 1°, for the network with 100 neurons representing 100°.
- We have shown how error that is accumulated due to imperfections of the robot (motor commands do not perfectly correspond to executed movements) can be corrected with external sensing, i.e., vision.
- We have demonstrated online learning of the reference pose in a closed behavioral loop, i.e., with the weight adaptation occurring in parallel to the robot's movements and path integration. Plastic weights are updated in timesteps, in which the learning conditions are fulfilled: the respective pre- and post-synaptic spikes co-occur in the same timestep. These updates can lead to one-shot learning (as shown here). The network can also be configured to require several co-activations of pre- and post-synaptic neurons for the updated weight to have a noticeable effect after the learning increment. We have shown how multiple objects can be stored in the network by adding one “label” neuron per object and a set of *N* plastic synapses, where *N* is the size of the head direction network layers (*N* = 100 here).
- We have introduced a number of structural motifs that solve computational tasks involved in path integration and map formation, that is, setting, resetting, and shifting connections and boosting and pre-shaping, as well as input and output interfaces between the non-spiking periphery and the neuromorphic chip.

When presenting the SNN model, we paid particular attention to the computing modules that realize important computational primitives that can be reused as building blocks in other tasks and on other neuromorphic hardware. Thus, we hope to contribute to building-up a neuromorphic “instruction set” that will allow us to design neuronal models, in particular for robotic tasks. Such neuronal models can be built using known biological neural circuits, creating a complementary approach to data-driven learning, which can be too costly in learning time and data-preparation effort for some applications. In comparison to previous work on neuromorphic head direction estimation, we evaluate system performance in a real-world task to estimate the 2D head pose of a humanoid iCub robot, particularly emphasizing the required interfaces between different hardware components. We have shown that disturbances can be mitigated by using information of different sensory modalities, and we evaluated how the path integration error relates to scaling of the network.

The main contributions of this work that we would like to emphasize are:

- We use “place code” to represent values (e.g., the angles of the head's pose): we represent values by the identity of the most active neuron (or localized region) in a neuronal population (layer). In particular, taking advantage of Loihi's precise nature, we use “one-hot” and “single-spike” encoding here, making every spike matter in our network.
- We show an example of combining rate code and place code to represent values in an SNN architecture, and we show how non-spiking sensory input can drive a spiking network.
- We use recurrent self-excitatory connections to create self-sustained activation in a neuron or neuronal population: an active neuron continues spiking to represent the current estimate of the pose in the SNN, even in the absence of input. This models the working memory of biological neural systems.
- We propose connectivity patterns between neuronal populations that solve different computational tasks:
  - *mapping* activity from one population to another one in a one-to-one or shifted manner;
  - *resetting* activity by inhibiting the currently active neuron(s) and activating another/others;
  - *boosting* the whole population through one-to-all connections;
  - providing subthreshold localized input (*preshape*) to create a potentiality for activation, i.e., when boosted, such a preshape can lead to fully-fledged activation.
- We demonstrate the integration of different modalities: input from one modality (motor command) is *integrated* into the network to produce the pose, and input from another modality (vision) is mapped onto the network through *plastic*—learned—synapses.
- Finally, we demonstrate one-shot online learning of the object-centering pose and how it can be used to generate object-directed gaze. To use the learned pose, we introduced an additional layer that can read out the learned pose without triggering a reset. Thus, we explicitly distinguish the “remembered” and the “currently perceived” object-centering pose representation, modeling different “directions of fit” from the theory of intentionality (Searle and Willis, 1983).

These elements form the basic algorithmic building blocks for pose estimation and SLAM-like systems in neuromorphic technologies. In this work, we realize the SNN to estimate the pose of a robot's head within two degrees of freedom (yaw-pitch). The error of the head-direction network compared to the integrated velocity commands in software remains below “one neuron” (i.e., an angle corresponding to *V*_*thr*_ from Equation 1). Plastic synaptic connections between the yaw-pitch motor space and visually-activated object-neurons in our SNN are learned to store the positions of objects autonomously during operation, i.e., showing online learning. These connections can be used to produce goal-directed head-movements toward stored poses, “looking back” at objects. The stored associations are also used to correct the pose, as the path integration process may be subject to drift (as shown in Figure 6).

This work also highlights the interfaces that we developed between the iCub robot and the Loihi chip. System integration is an important challenge in robotics in general and in neuromorphic robotics in particular. Our solution is still in a prototype stage but already achieves real-time performance (processing loop of <10 ms). Tighter integration of the hardware system will further improve the system's latency. When combined with a more powerful object recognition system, our pose estimation and learning SNN can be used as a component of an interactive scene representation system for robotic and augmented reality applications.

## Data Availability Statement

All datasets generated for this study are included in the article.

## Author Contributions

RK, AR, and VL have worked on the SNN on chip, run the simulated experiments, and worked on SNN-related plots and results. BS helped with the initial experiments and writing. CB supervised the work related to iCub robot and event-based vision and helped with writing. AG planned and performed the experiments with the robot, developed the software infrastructure required to connect the robot and neuromorphic hardware, worked on the plots and text related to robot experiments. YS conceptualized the study, suggested the experiments, supervised all work, and put it in the context of SoA.

## Conflict of Interest

The authors declare that the research was conducted in the absence of any commercial or financial relationships that could be construed as a potential conflict of interest.

## Footnotes

^1^https://services.ini.uzh.ch/permlink.php/puGu3hai

## References

[B1] Alzugaray I. Chli M. (2018). Asynchronous corner detection and tracking for event cameras in real-time. IEEE Robot. Autom. Lett. 3, 3177–3184. 10.1109/LRA.2018.2849882

[B2] Arena P. Maceo S. Patane L. Strauss R. (2013). A spiking network for spatial memory formation: towards a fly-inspired ellipsoid body model, in The 2013 International Joint Conference on Neural Networks (IJCNN) (Dallas, TX: IEEE), 1–6. 10.1109/IJCNN.2013.6706882

[B3] Arleo A. Gerstner W. (2000). Spatial cognition and neuro-mimetic navigation: a model of hippocampal place cell activity. Biol Cybern. 83, 287–299. 10.1007/s004220000171 11007302

[B4] Barrera A. Weitzenfeld A. (2008). Biologically-inspired robot spatial cognition based on rat neurophysiological studies. Auton. Robots 25, 147–169. 10.1007/s10514-007-9074-3

[B5] Bartolozzi C. Rea F. Clercq C. Fasnacht D. B. Indiveri G. Hofstätter M. (2011). Embedded neuromorphic vision for humanoid robots, in IEEE Computer Society Conference on Computer Vision and Pattern Recognition Workshops (Colorado Springs, CO). 10.1109/CVPRW.2011.5981834

[B6] Bell C. Storck T. Sandamirskaya Y. (2014). Learning to look: a dynamic neural fields architecture for gaze shift generation, in ICANN (Hamburg), 699–706. 10.1007/978-3-319-11179-7_88

[B7] Burak Y. Fiete I. R. (2009). Accurate path integration in continuous attractor network models of grid cells. PLoS Comput. Biol. 5:e1000291. 10.1371/journal.pcbi.1000291 19229307 PMC2632741

[B8] Chen C. Seff A. Kornhauser A. Xiao J. (2015). DeepDriving: learning affordance for direct perception in autonomous driving, in 2015 IEEE International Conference on Computer Vision (ICCV) (Santiago). 10.1109/ICCV.2015.312

[B9] Conklin J. Eliasmith C. (2005). A controlled attractor network model of path integration in the rat. J. Comput. Neurosci. 18, 183–203. 10.1007/s10827-005-6558-z 15714269

[B10] Corradi F. Zambrano D. Raglianti M. Passetti G. Laschi C. Indiveri G. (2014). Towards a neuromorphic vestibular system. IEEE Trans. Biomed. Circuits Syst. 8, 669–680. 10.1109/TBCAS.2014.2358493 25314706

[B11] Cuperlier N. Quoy M. Gaussier P. (2007). Neurobiologically inspired mobile robot navigation and planning. Front. Neurorobot. 1:3. 10.3389/neuro.12.003.2007 18958274 PMC2533588

[B12] Dalgaty T. Vianello E. De Salvo B. Casas J. (2018). Insect-inspired neuromorphic computing. Curr. Opin. Insect Sci. 30, 59–66. 10.1016/j.cois.2018.09.006 30553486

[B13] Davies M. Srinivasa N. Lin T. H. Chinya G. Cao Y. Choday S. H. (2018). Loihi: a neuromorphic manycore processor with on-chip learning. IEEE Micro 38, 82–89. 10.1109/MM.2018.112130359

[B14] Edvardsen V. (2017). Long-range navigation by path integration and decoding of grid cells in a neural network, in 2017 International Joint Conference on Neural Networks (IJCNN) (Anchorage, AK: IEEE), 4348–4355.

[B15] Engelhard N. Endres F. Hess J. Sturm J. Burgard W. (2011). Real-time 3D visual slam with a hand-held RGB-D camera, in Proceedings of the RGB-D Workshop on 3D Perception in Robotics at the European Robotics Forum (Vasteras), Vol. 180, 1–15.

[B16] Erdem U. M. Milford M. J. Hasselmo M. E. (2015). A hierarchical model of goal directed navigation selects trajectories in a visual environment. Neurobiol. Learn. Mem. 117, 109–121. 10.1016/j.nlm.2014.07.003 25079451

[B17] Fisher Y. E. Lu J. D'Alessandro I. Wilson R. I. (2019). Sensorimotor experience remaps visual input to a heading-direction network. Nature 576, 121–125. 10.1038/s41586-019-1772-4 31748749 PMC7753972

[B18] Furber S. B. Lester D. R. Plana L. A. Garside J. D. Painkras E. Temple S. (2012). Overview of the SpiNNaker system architecture. IEEE Trans. Comput. 62, 2454–2467. 10.1109/TC.2012.142

[B19] Gallego G. Delbruck T. Orchard G. Bartolozzi C. Taba B. Censi A. (2019). Event-based vision: a survey. arxiv [Preprint] arXiv 1904.08405. 10.1109/TPAMI.2020.3008413 32750812

[B20] Galluppi F. Lagorce X. Stromatias E. Pfeiffer M. Plana L. A. Furber S. B.. (2015). A framework for plasticity implementation on the spinnaker neural architecture. Front. Neurosci. 8:429. 10.3389/fnins.2014.00429 25653580 PMC4299433

[B21] Gehrig M. Shrestha S. B. Mouritzen D. Scaramuzza D. (2020). Event-based angular velocity regression with spiking networks. arXiv 2003.02790.

[B22] Goodridge J. P. Touretzky D. S. (2000). Modeling attractor deformation in the rodent head-direction system. J. Neurophysiol. 83, 3402–3410. 10.1152/jn.2000.83.6.3402 10848558

[B23] Green J. Adachi A. Shah K. K. Hirokawa J. D. Magani P. S. Maimon G. (2017). A neural circuit architecture for angular integration in Drosophila. Nature 546:101. 10.1038/nature22343 28538731 PMC6320684

[B24] Grossberg S. (1988). Nonlinear neural networks: principles, mechanisms, and architectures. Neural Netw. 1, 17–61. 18579344

[B25] Gu T. Yan R. (2019). An improved loop closure detection for RatSLAM, in 2019 5th International Conference on Control, Automation and Robotics (ICCAR) (Beijing: IEEE), 884–888.

[B26] Hahnloser R. H. (2003). Emergence of neural integration in the head-direction system by visual supervision. Neuroscience 120, 877–891. 10.1016/S0306-4522(03)00201-X 12895528

[B27] Heinze S. (2017). Unraveling the neural basis of insect navigation. Curr. Opin. Insect Sci. 24, 58–67. 10.1016/j.cois.2017.09.001 29208224 PMC6186168

[B28] Heinze S. Narendra A. Cheung A. (2018). Principles of insect path integration. Curr. Biol. 28, R1043–R1058. 10.1016/j.cub.2018.04.058 30205054 PMC6462409

[B29] Honkanen A. Adden A. Da Silva Freitas J. Heinze S. (2019). The insect central complex and the neural basis of navigational strategies. J. Exp. Biol. 222:jeb188854. 10.1242/jeb.188854 30728235 PMC6474401

[B30] Indiveri G. Chicca E. Douglas R. J. (2009). Artificial cognitive systems: from VLSI networks of spiking neurons to neuromorphic cognition. Cogn. Comput. 1, 119–127. 10.1007/s12559-008-9003-623853161

[B31] Jauffret A. Cuperlier N. Gaussier P. (2015). From grid cells and visual place cells to multimodal place cell: a new robotic architecture. Front. Neurorobot. 9:1. 10.3389/fnbot.2015.00001 25904862 PMC4388131

[B32] Kreiser R. Aathmani D. Qiao N. Indiveri G. Sandamirskaya Y. (2018a). Organizing sequential memory in a neuromorphic device using dynamic neural fields. Front. Neurosci. 12:717. 10.3389/fnins.2018.00717 30524218 PMC6262404

[B33] Kreiser R. Cartiglia M. Martel J. N. Conradt J. Sandamirskaya Y. (2018b). A neuromorphic approach to path integration: a head-direction spiking neural network with vision-driven reset, in 2018 IEEE International Symposium on Circuits and Systems (ISCAS) (Florence). 10.1109/ISCAS.2018.8351509

[B34] Kreiser R. Pienroj P. Renner A. Sandamirskaya Y. (2018c). Pose estimation and map formation with spiking neural networks: towards neuromorphic SLAM, in IEEE/RSJ International Conference on Intelligent Robots and Systems (Madrid). 10.1109/IROS.2018.8594228

[B35] Kreiser R. Renner A. Sandamirskaya Y. (2019a). Error-driven learning for self-calibration in a neuromorphic path integration system, in Robust AI for Neurorbotics (Edinburgh).

[B36] Kreiser R. Waibel G. Renner A. Sandamirskaya Y. (2019b). Self-calibration and learning on chip: towards neuromorphic robots, in IEEE/RSJ International Conference on Intelligent Robots and Systems (IROS), Breaking News (Macau).

[B37] Krichmar J. L. Wagatsuma H. (2011). Neuromorphic and Brain-Based Robots, Vol. 233 Cambridge: Cambridge University Press.

[B38] Liang D. Kreiser R. Nielsen C. Qiao N. Sandamirskaya Y. Indiveri G. (2019). Neural state machines for robust learning and control of neuromorphic agents. IEEE J. Emerg. Select. Top. Circuits Syst. 9, 679–689. 10.1109/JETCAS.2019.2951442

[B39] Massoud T. M. Horiuchi T. K. (2011a). A neuromorphic VLSI head direction cell system. IEEE Trans. Circuits Syst. I Reg. Pap. 58, 150–163. 10.1109/TCSI.2010.2055310

[B40] Massoud T. M. Horiuchi T. K. (2011b). Online correction of orientation estimates using spatial memory in a neuromorphic head direction system, in Proceedings–IEEE International Symposium on Circuits and Systems (Rio de Janeiro). 10.1109/ISCAS.2011.5938094

[B41] Massoud T. M. Horiuchi T. K. (2012). A neuromorphic VLSI grid cell system, in ISCAS 2012–2012 IEEE International Symposium on Circuits and Systems. (Seoul). 10.1109/ISCAS.2012.6271787

[B42] Merolla P. A. Arthur J. V. Alvarez-Icaza R. Cassidy A. S. Sawada J. Akopyan F.. (2014). Artificial brains. A million spiking-neuron integrated circuit with a scalable communication network and interface. Science 345, 668–673. 10.1126/science.1254642 25104385

[B43] Metta G. Fitzpatrick P. Natale L. (2006). YARP: yet another robot platform. Int. J. Adv. Robot. Syst. 3, 43–48. 10.5772/5761

[B44] Metta G. Sandini G. Vernon D. Natale L. Nori F. (2008). The iCub humanoid robot: An open platform for research in embodied cognition, in Performance Metrics for Intelligent Systems (PerMIS) Workshop (Gaithersburg, MD), 50–56. 10.1145/1774674.1774683

[B45] Milford M. Schulz R. (2014). Principles of goal-directed spatial robot navigation in biomimetic models. Philos. Trans. R. Soc. B Biol. Sci. 369, 1–13. 10.1098/rstb.2013.0484 25267826 PMC4186237

[B46] Milford M. J. Wyeth G. F. Prasser D. (2004). RatSLAM: a hippocampal model for simultaneous localization and mapping, in Proceeding of the 2004 IEEE international Conference on Robotics & *Automation* (New Orleans, LA), 403–408. 10.1109/ROBOT.2004.1307183

[B47] Mnih V. Kavukcuoglu K. Silver D. Rusu A. a Veness J.. (2015). Human-level control through deep reinforcement learning. Nature 518, 529–533. 10.1038/nature14236 25719670

[B48] Moradi S. Qiao N. Stefanini F. Indiveri G. (2018). A scalable multicore architecture with heterogeneous memory structures for dynamic neuromorphic asynchronous processors (DYNAPs). IEEE Trans. Biomed. Circuits Syst. 12, 106–122. 10.1109/TBCAS.2017.2759700 29377800

[B49] Neftci E. O. Mostafa H. Zenke F. (2019). Surrogate gradient learning in spiking neural networks. IEEE Signal Process. Mag. 36, 51–63. 10.1109/MSP.2019.2931595

[B50] Pfeiffer K. Homberg U. (2014). Organization and functional roles of the central complex in the insect brain. Annu. Rev. Entomol. 59, 165–184. 10.1146/annurev-ento-011613-162031 24160424

[B51] Posch C. Matolin D. Wohlgenannt R. (2008). An asynchronous time-based image sensor, in 2008 IEEE International Symposium on Circuits and Systems (Seattle, WA), 2130–2133. 10.1109/ISCAS.2008.4541871

[B52] Qiao N. Mostafa H. Corradi F. Osswald M. Stefanini F. Sumislawska D.. (2015). A reconfigurable on-line learning spiking neuromorphic processor comprising 256 neurons and 128K synapses. Front. Neurosci. 9:141. 10.3389/fnins.2015.00141 25972778 PMC4413675

[B53] Redish A. Elga A. Touretzky D. (1996). A coupled attractor model of the rodent head direction system. Netw. Comput. Neural Syst. 7, 671–685. 10.1088/0954-898x/7/4/004

[B54] Samu D. Erős P. Ujfalussy B. Kiss T. (2009). Robust path integration in the entorhinal grid cell system with hippocampal feed-back. Biol. Cybern. 101, 19–34. 10.1007/s00422-009-0311-z 19381679

[B55] Sandamirskaya Y. Storck T. (2014). Neural-dynamic architecture for looking: shift from visual to motor target representation for memory saccade, in ICDL-EPIROB (Genoa). 10.1109/DEVLRN.2014.6982951

[B56] Sandamirskaya Y. Storck T. (2015). Chapter: learning to look and looking to remember: a neural-dynamic embodied model for generation of saccadic gaze shifts and memory formation, in Artificial Neural Network, Vol. 4, eds Koprinkova-Hristova P. Mladenov V. Kasabov N. K. (Springer), 175–200. 10.1007/978-3-319-09903-3_9

[B57] Scaramuzza D. Fraundorfer F. (2011). Visual odometry. IEEE Robot. Autom. Mag. 18, 80–92. 10.1109/MRA.2011.943233

[B58] Schemmel J. Brüderle D. Grübl A. Hock M. Meier K. Millner S. (2010). A wafer-scale neuromorphic hardware system for large-scale neural modeling, in ISCAS 2010–2010 IEEE International Symposium on Circuits and Systems: Nano-Bio Circuit Fabrics and Systems (Paris), 1947–1950. 10.1109/ISCAS.2010.5536970

[B59] Searle J. R. Willis S. (1983). Intentionality: An Essay in the Philosophy of Mind. Cambridge: Cambridge University Press.

[B60] Seelig J. D. Jayaraman V. (2013). Feature detection and orientation tuning in the Drosophila central complex. Nature 503, 262–266. 10.1038/nature12601 24107996 PMC3830704

[B61] Seelig J. D. Jayaraman V. (2015). Neural dynamics for landmark orientation and angular path integration. Nature 521, 186–191. 10.1038/nature14446 25971509 PMC4704792

[B62] Shrestha S. B. Orchard G. (2018). Slayer: spike layer error reassignment in time, in Advances in Neural Information Processing Systems (Montreal, QC), 1412–1421.

[B63] Skaggs W. E. Knierim J. J. Kudrimoti H. S. McNaughton B. L. (1995). A model of the neural basis of the rat's sense of direction, in Advances in Neural Information Processing Systems (Denver, CO), 173–180. 11539168

[B64] Song P. Wang X. J. (2005). Angular path integration by moving “hill of activity”: a spiking neuron model without recurrent excitation of the head-direction system. J. Neurosci. 25, 1002–1014. 10.1523/JNEUROSCI.4172-04.2005 15673682 PMC6725619

[B65] Stachniss C. Leonard J. J. Thrun S. (2016). Simultaneous localization and mapping, in Springer Handbook of Robotics, eds Siciliano B. Khatib O. (Springer), 1153–1176. 10.1007/978-3-319-32552-1_46

[B66] Stratton P. Wyeth G. Wiles J. (2010). Calibration of the head direction network: a role for symmetric angular head velocity cells. J. Comput. Neurosci. 28, 527–538. 10.1007/s10827-010-0234-7 20354898

[B67] Stringer S. M. Trappenberg T. P. Rolls E. T. De Araujo I. E. (2002). Self-organizing continuous attractor networks and path integration: one-dimensional models of head direction cells. Netw. Comput. Neural Syst. 13, 217–242. 10.1088/0954-898X/13/2/304 12061421

[B68] Tang G. Shah A. Michmizos K. P. (2019). Spiking neural network on neuromorphic hardware for energy-efficient unidimensional slam. arXiv 1903.02504. 10.1109/IROS40897.2019.8967864

[B69] Taube J. S. (2007). The head direction signal: origins and sensory-motor integration. Annu. Rev. Neurosci. 30, 181–207. 10.1146/annurev.neuro.29.051605.112854 17341158

[B70] Thrun S. Montemerlo M. Dahlkamp H. Stavens D. Aron A. Diebel J. (2007). Stanley: the robot that won the DARPA grand challenge, in Springer Tracts in Advanced Robotics, eds Buehler M. Iagnemma K. Singh S. (Springer), 661–692. 10.1007/978-3-540-73429-1_1

[B71] Turner-Evans D. Wegener S. Rouault H. Franconville R. Wolff T. Seelig J. D.. (2017). Angular velocity integration in a fly heading circuit. eLife 6:e23496. 10.7554/eLife.23496 28530551 PMC5440168

[B72] Turner-Evans D. B. Jayaraman V. (2016). The insect central complex. Curr. Biol. 26, R445–R460. 10.1016/j.cub.2016.04.006 27269718

[B73] Weikersdorfer D. Hoffmann R. Conradt J. (2013). Simultaneous localization and mapping for event-based vision systems, in International Conference on Computer Vision Systems (Thessaloniki: Springer), 133–142. 10.1007/978-3-642-39402-7_14

[B74] Yu F. Shang J. Hu Y. Milford M. (2019). NeuroSLAM: a brain-inspired SLAM system for 3D environments. Biol. Cybern. 113, 515–545. 10.1007/s00422-019-00806-9 31571007

[B75] Zhang H. Tang H. Yan R. (2019). Multi-sensor fusion for a brain-inspired SLAM system, in 2019 5th International Conference on Control, Automation and Robotics (ICCAR) (Beijing: IEEE), 619–623.

